# Comparison of human erythrocyte purine nucleotide metabolism and blood purine and pyrimidine degradation product concentrations before and after acute exercise in trained and sedentary subjects

**DOI:** 10.1007/s12576-017-0536-x

**Published:** 2017-04-21

**Authors:** Wioleta Dudzinska, M. Suska, A. Lubkowska, K. Jakubowska, M. Olszewska, K. Safranow, D. Chlubek

**Affiliations:** 1 Department of Physiology, Faculty of Biology, University of Szczecin, 3c Felczaka St., 71-412 Szczecin, Poland; 2 Department of Functional Diagnostics and Physical Medicine, Faculty of Health Sciences, Pomeranian Medical University in Szczecin, Szczecin, Poland; 3 Department of Biochemistry and Medical Chemistry, Pomeranian Medical University in Szczecin, Szczecin, Poland

**Keywords:** Exercise, Trained, Sedentary, Purine and pyrimidine nucleotides, Uridine, Erythrocyte, VO_2max_, APRT, HGPRT

## Abstract

This study aimed at evaluating the concentration of erythrocyte purine nucleotides (ATP, ADP, AMP, IMP) in trained and sedentary subjects before and after maximal physical exercise together with measuring the activity of purine metabolism enzymes as well as the concentration of purine (hypoxanthine, xanthine, uric acid) and pyrimidine (uridine) degradation products in blood. The study included 15 male elite rowers [mean age 24.3 ± 2.56 years; maximal oxygen uptake (VO_2max_) 52.8 ± 4.54 mL/kg/min; endurance and strength training 8.2 ± 0.33 h per week for 6.4 ± 2.52 years] and 15 sedentary control subjects (mean age 23.1 ± 3.41 years; VO_2max_ 43.2 ± 5.20 mL/kg/min). Progressive incremental exercise testing until refusal to continue exercising was conducted on a bicycle ergometer. The concentrations of ATP, ADP, AMP, IMP and the activities of adenine phosphoribosyltransferase (APRT), hypoxanthine-guanine phosphoribosyltransferase (HGPRT) and phosphoribosyl pyrophosphate synthetase (PRPP-S) were determined in erythrocytes. The concentrations of hypoxanthine, xanthine, uric acid and uridine were determined in the whole blood before exercise, after exercise, and 30 min after exercise testing. The study demonstrated a significantly higher concentration of ATP in the erythrocytes of trained subjects which, in part, may be explained by higher metabolic activity on the purine re-synthesis pathway (significantly higher PRPP-S, APRT and HGPRT activities). The ATP concentration, just as the ATP/ADP ratio, as well as an exercise-induced increase in this ratio, correlates with the VO_2max_ level in these subjects which allows them to be considered as the important factors characterising physical capacity and exercise tolerance. Maximal physical exercise in the group of trained subjects results not only in a lower post-exercise increase in the concentration of hypoxanthine, xanthine and uric acid but also in that of uridine. This indicates the possibility of performing high-intensity work with a lower loss of not only purine but also pyrimidine.

## Introduction

In erythrocytes, the adenylate pool size (ATP + ADP + AMP) depends on the balance between AMP synthesis and degradation. AMP is related to the other pool components via the adenylate kinase reaction (2ADP↔ATP + AMP) [1]. There are two pathways whereby AMP is synthesized in erythrocytes: from adenine and adenosine. The salvage of adenine depends upon two sequential enzymatic reactions. The first synthesizes 5-phosphoribosyl pyrophosphate (PRPP) from ribose-5-phosphate and ATP in the presence of PRPP synthetase (PRPP-S; EC 2.7.6.1); in the second, adenine phosphoribosyltransferase (APRT; EC 2.4.2.7) catalyses the formation of AMP from PRPP and adenine [2]. On the other hand, adenosine kinase (EC 2.7.1.20) catalyses the salvage synthesis of AMP from adenosine and ATP. Besides adenine and adenosine, hypoxanthine (Hyp) is usually considered a major substrate of salvage pathways. Under normal conditions, up to 90% of Hyp, which is a breakdown product of ATP, is subject to reutilisation to IMP [3]. This reaction takes place in the presence of PRPP and is catalysed by hypoxanthine-guanine phosphoribosyltransferase (HGPRT; EC 2.4.2.8).

Physical exercise, particularly of high intensity, contributes to a decreased concentration of oxygen in relation to its demand, leading to bioenergetic hypoxia of muscles, the result of which is increased ATP degradation, being accompanied by IMP and NH_3_ accumulation [4, 5]. Most IMP is re-synthesised to ATP during recovery in the purine nucleotide cycle, but part of IMP is dephosphorylated to a purine basis inosine (Ino), and then to Hyp [4]. The products of IMP degradation, particularly Hyp, may again be included in the IMP pool in the reaction being catalysed by HGPRT (purine salvage pathway) or, after leaving the muscle, may accumulate in blood plasma [6–9]. Owing to membrane transporters, nucleosides and purine bases (mainly Hyp) are in the balance between blood plasma and erythrocytes, for which they constitute a substrate in salvage-type reactions [2].

In addition, one of the permanent phenomena accompanying intense physical exercises is metabolic acidosis and hyperphosphatemia. The higher an increase in the hydrogen ion concentration, the higher the exercise intensity. A fall in pH in red blood cells results in a decrease in 2,3-BPG and ADP (allosteric inhibitors of PRPP-S) with a concomitant increase in intracellular inorganic phosphate—Pi (PRPP-S activator) and ATP [10]. Thus, an increase in Pi concentration in blood together with a decrease in pH could play a supportive role in the activation of PRPP-S because this enzyme has an absolute requirement for Pi for its activation [10, 11]. Therefore, exercise could favour increased synthesis of PRPP, a co-substrate in the reactions being catalysed by APRT and HGPRT and, in consequence, result in an increase in the metabolic activity of purines on the salvage pathway. There is some support in the literature for the hypothesis being made. An in vitro study on the effect of Pi on purine nucleotide concentration showed that an increase occurred in IMP concentration in red blood cells being incubated in a solution containing 2 mmol/L Pi (more than the physiological concentration) [10, 12]; the in vitro study demonstrated that the uptake of Hyp and accumulation of IMP in red blood cells is significantly intensified at acidic pH, high external phosphate concentrations, and low pO_2_, i.e. under the conditions which accompany intense physical exercise. They suggested that erythrocytes could play a role in the removal of Hyp from anoxic tissue. In addition, the enhanced synthesis of PRPP explains the increased levels of ATP and IMP found in the erythrocytes of patients with chronic renal failure, in which blood pH is decreased and plasma Pi concentrations are increased [13–15].

Thus, a post-exercise increase in the concentration of purine re-synthesis substrates (mainly Hyp), and an increase in the concentration of plasma phosphate, especially in combination with acidosis, may lead to changes in the activity of enzymes being involved in purine metabolism and, in consequence, in their concentrations. Therefore, there are reasons to evaluate the effect of high-intensity physical exercise on the concentration of purine nucleotides in red blood cells. This is increasingly true in that there are limited data so far on the post-exercise changes in erythrocyte purine concentration; they are not very consistent and refer mainly to evaluation of the effect of physical exercise on the concentration of adenine nucleotides in the red blood cells of untrained subjects. In these studies, some authors report an increase in ATP concentration [16], whereas other ones show no changes in ATP concentration [17, 18]. Those differences are probably due to different protocols, i.e. different characteristics of subjects (age, physical activity, health), different training and tests (type, intensity, duration of exercise tests), various methods of measuring the purine concentration, etc. The absence of studies investigating the effect of physical exercise on purine metabolism in the erythrocytes of trained subjects is surprising, in view of the fact that training leads to many adaptive changes, also in blood and the erythrocyte system [19]. Training enhances O_2_ flux to the working muscles at all levels of regulation: it increases maximal cardiac output, improves blood flow to the muscles by stimulating vascularisation, and improves the rheological properties of red blood cells. Training increases total haemoglobin mass which increases the amount of O_2_ that can be carried by blood. It also increases red blood cell 2,3-BPG, which increases the sensitivity of Hb–O_2_ affinity to acidification-dependent O_2_ release [19]. It was also shown that adaptation to physical exercise in trained subjects is associated with the improvement of potential for re-phosphorylation of Hyp to IMP through increased HGPRT activity in the muscles [20].

Given the above, this study involved evaluation of the concentration of purine nucleotides (ATP, ADP, AMP, IMP) in the erythrocytes of trained and sedentary subjects before and after maximal physical exercise together with measuring the activity of purine metabolism enzymes being under the regulatory effect of pH and Pi. Furthermore, apart from evaluation of resting and post-exercise changes in the concentration of purine degradation products in blood, i.e. Hyp, xanthine (Xa) and uric acid (UA), changes in the concentration of uridine (Urd), which is a product of pyrimidine degradation, was measured. To our knowledge, this is the first study in this area.

One of the main effects of regular physical activity is increased physical capacity, a measure of which is the magnitude of maximal oxygen uptake (VO_2max_). It is now accepted that the VO_2max_ during whole-body exercise in humans is not constrained by the capacity of mitochondrial oxygen consumption but by the magnitude of oxygen delivery to the working muscles [21, 22]. A recent study has shown that erythrocyte ATP concentration and changes in its level may regulate the magnitude of oxygen delivery to tissues [23]. Therefore, the study evaluated a relationship between the VO_2max_ level and the ATP concentration.

## Materials and methods

### Subjects

The study included 15 male elite rowers (mean age 24.3 ± 2.56 years, endurance and strength training 8.2 ± 0.33 h per week for 6.4 ± 2.52 years) and 15 sedentary control subjects (mean age 23.1 ± 3.41 years).

A typical training program for the rowers comprised rowing at different energy expenditure levels in addition to one or two weight-lifting sessions. In the group of rowers, examinations were conducted at the end of the preparatory period.

The control subjects performed normal daily activities and had never participated in regular endurance training; for at least the previous 2 years, they had not been involved in any competitive or recreational sports.

All subjects were healthy and normotensive, with a body mass index (BMI) between 21.5 ± 2.51 kg/m^2^ (trained) and 22.5 ± 2.32 kg/m^2^ (sedentary). Anthropometric and physiological characteristics of the participants are presented in Table 1. They had no history of any metabolic and cardiovascular diseases. The participants were nonsmokers and refrained from taking any medications or supplements known to affect metabolism.

**Table 1:** Baseline characteristics of sedentary and trained subjects

	Sedentary group	Trained group
Number	15	15
Age (years)	23.1 ± 3.41	24.3 ± 2.56
BMI (kg/m^2^)	22.5 ± 2.32	21.5 ± 2.51
HRrest (bpm)	63.1 ± 7.51	59.0 ± 6.33
SBP (mmHg)	113.4 ± 9.24	117.6 ± 8.32
DBP (mmHg)	72.5 ± 6.11	68.2 ± 7.21
Duration of training (years)	–	6.4 ± 2.52
Training time (hours per week)	–	8.2 ± 0.33
Time of exercise test (min)	19.3 ± 4.55	23.0 ± 5.97
VO_2max_ (mL/kg/min)	43.0 ± 5.20	52.8 ± 4.54**
2,3-BPG (µmol/mL)	1.85 ± 0.21	2.3 ± 0.18*

Values are given as mean ± SD

*BMI* body mass index, *HR* heart rate, *SBP* systolic blood pressure, *DBP* diastolic blood pressure, *VO*_*2max*_ maximal oxygen uptake, *2,3-BPG* 2,3-bisphosphoglycerate

* *P* < 0.01; ** *P* < 0.001; different from sedentary

Participants were volunteers and were informed about the study protocol, the risks of all tests, their rights, and they gave their written informed consent before the initiation of the experiment. The study was approved by the local ethics committee (Ethics Committee of Pomeranian Medical University) in accordance with the Helsinki Declaration.

Exercise testing was performed at the laboratory of the Department of Physiology, Faculty of Biology, University of Szczecin, Poland, in the early-morning hours. The subjects reported to the laboratory a few hours after having a light breakfast (without tea and coffee).

### Anthropometric measurements

Body height (cm) was measured to the nearest centimeter using a rigid stadiometer. Body mass (kg) was measured in underwear to the nearest 0.1 kg using an electronic scale. BMI was calculated by dividing weight in kilograms by height in square meters (kg/m^2^).

### Blood pressure

Systolic and diastolic blood pressure (SBP and DBP) were measured according to guidelines at the right arm after a 10-min rest by using a calibrated sphygmomanometer.

### Experimental protocol

The participants were subjected to tests of maximal physical capacity, establishing their maximal oxygen uptake (VO_2max_) using a direct method. In order to estimate VO_2max_, a progressive ergocycle test was applied. The exercise was preceded by a 5-min warm-up (25 W). The test was performed on a bicycle ergometer (Kettler X-7, Germany).

The proper test began at 70 W while maintaining 70 revolutions per minute. The effort continued with an increasing load (20 W every 3 min) until refusal, or until a tested individual was not able to maintain the required rotation frequency [24].

During the exercise, oxygen uptake (VO_2_) was measured continuously using an Oxycon gas analyzer (Jaeger, Germany). Heart rate was measured with a Polar sport tester.

### Sample collection

Venous blood was taken from the antecubital forearm vein before exercise, after exercise, and 30 min after exercise testing. Blood samples were taken into two separate tubes (Monovette^®^, Sarstedt, Germany). The first tube consisted of lithium heparinate (15 IU/mL blood, 4.9 mL) and the second one (which was used for detrermination of purines and pyrimidines) of sodium fluoride and lithium heparin (1 mg/mL blood + 16 IU/mL, 2.7 mL).

Both concentration of tested compounds and activity of enzimes were determined in whole blood, red blood cells and plasma. Thus, later procedures were carried out according to specific methods of determination. They are presented in the section below.

### Blood analysis

Venous blood gases were determined by using a blood gas-pH analyzer (Ciba Corning 248 blood gas-pH analyzer, Medford, MA, USA).

Inorganic phosphorous in plasma was determined using a Randox Laboratory diagnostic kit REF PH1016 (Randox Laboratories Ltd. Co. Antrim, UK).

Lactate concentration was determined using a Dr Lange Lp-20 analytical kit (Lange, Germany).

To measure 2,3-BPG concentration in red blood cells, the ultraviolet enzymatic method (Kit 35-UV, Sigma, St. Louis, MO, USA) was used.

### Determination of purines and pyrimidines

The purine nucleotide (ATP, ADP, AMP, IMP) concentrations were determined in erythrocytes. The Hyp, Xan, UA and Urd concentrations were determined in the whole blood. The purine and pyrimidine concentrations being analysed were determined by high-performance liquid chromatography (HPLC) according to the method used by Smolenski et al. [25].

In order to isolate erythrocytes, blood samples were centrifuged within 5 min from taking a sample (650×*g*, 10 min, 4 °C). The plasma and buffy coat were removed, and the erythrocytes were washed 3 times in HEPES buffered Krebs–Ringer solution, containing 125 mmol/L NaCl, 2.7 mmol/L KCl, 1.2 mmol/L MgCl_2_, 1.2 mmol/L KH_2_PO_4_, and 5 mmol/L glucose.

The samples (500 μL) of heparinised blood or erythrocytes were deproteinised with an equal volume of 1.3 mol/L HClO_4_, mixed, and then centrifuged at 20,000×*g* for 5 min at 4 °C. The supernatant (400 μL) was neutralised with 130–160 μL of 1 mol/L K_3_PO_4_ (to pH 5–7). The samples were centrifuged again under the same conditions as previously, and aliquots of 100 µL were injected into the sample loop. Purines were separated using a gradient elution system (buffer A: 150 mmol/L KH_2_PO_4_/K_2_HPO_4_, 150 mmol/L KCl, pH 6.0; buffer B: 15% solution of acetonitrile in buffer A) at a flow rate of 1 mL/min. Peaks were detected by absorbance at 254 nm. Chromatographic analysis was performed using a Hewlett–Packard Series 1050/1100 chromatograph.

The concentrations of nucleotides being determined were expressed in relation to erythrocyte volume. The isolated and washed erythrocytes were collected in Modulohm glass capillaries (volume 20 µL, length 75 mm). Hematocrit values were determined in duplicate by standard microhematocrit method and expressed as a percentage. The intra-erythrocyte concentrations of ATP, ADP, AMP and IMP are expressed as μmol/L red blood cell (RBC). The concentrations of Hyp, Xan, UA and Urd, being present in both erythrocytes and plasma, are expressed as μmol/L whole blood.

The values of total adenine nucleotide pool (TAN) and adenylate energy charge (AEC) were also calculated [2]:

$$\begin{aligned} {\text{TAN }} & = {\text{ ATP}} + {\text{ADP}} + {\text{AMP}} \\ {\text{AEC }} & = \frac{{[{\text{ATP}}] + 0.5 [ {\text{ADP}}]}}{{[{\text{ATP}}] + [{\text{ADP}}] + [{\text{AMP}}]}}. \\ \end{aligned}$$

### Determination of erythrocyte APRT and HGPRT activity

The activity of APRT and HGPRT was determined by HPLC in an erythrocyte lysate according to Sakuma et al. [26] and Rylance et al. [27].

The erythrocytes were separated by centrifugation at 1500×*g* for 10 min at 4 °C, washed 3 times with 0.9% NaCl and frozen at −80 °C.

100 μL of lysate was diluted with 500 μL of cold charcoal-dextran suspension containing 3.0 g/L charcoal and 0.3 g/L dextran in cold distilled water. After mixing for about 10 s in a vortex mixer, the lysates were left for 15 min at 4 °C, then centrifuged at 10 000×*g* for 15 min at 4 °C. The supernatants were used as samples for enzyme activity determination.

In the erythrocyte lysates, hemoglobin concentrations (Hb) was determined by the Drabkin’s method.

The enzyme reactions were started by adding 25 μL of erythrocyte lysate to the substrate mixture containing: 250 μL reagent A (100 mmol/L Tris HCl pH 7.4, 12 mmol/L MgCl_2_, 2 mmol/L Hyp, 0.4 mmol/L adenine) and 250 μL 2 mmol/L PRPP. After 5 min of incubation at 37^◦^C, a 200-μL sample was collected and placed in an Eppendorf tube containing 200 μL of 1.3 mol/L HClO_4_. After further 25 min of incubation, another 200 μL sample was collected and also placed in an Eppendorf tube containing HClO_4_. The samples were centrifuged (14,000×*g*, 5 min, 4 °C). The supernatant (200 μL) was neutralized (pH 5–7) with 1 mol/L K_3_PO_4_. The samples were centrifuged again (under the same conditions as previously) and 100 μL was taken for HPLC analysis [25].

The APRT activity was calculated from the increase of AMP + ADP + ATP between samples taken at 5 and 30 min of incubation and expressed as nmol/min/mg Hb.

The HPRT activity was calculated from the increase of IMP between samples taken at 5 and 30 min of incubation and expressed as nmol/min/mg Hb.

### Determination of erythrocyte PRPP-S activity

The erythrocytes were separated by centrifugation at 1500×*g* for 10 min at 4 °C. The received pellet was washed twice with PBS and then frozen and thawed two times, resuspended in 1 mL of ice-cold deionized water and used directly for PRPP-S quantification.

Measurement of the PRPP-S activity was performed using a Precice^®^ kit (Novocib, Lyon, France). The assay is based on a reaction in which, in the presence of ATP and P-ribose, PRPP-synthetase catalyzes the formation of PRPP. In the presence of Hyp, PRPP is converted to IMP by hypoxanthine-guanine phosphoribosyltransferase. IMP is immediately oxidized by a highly active IMP dehydrogenase in the presence of NAD with simultaneous formation of NADH_2_ being directly monitored spectrophotometrically at 340 nm. Assay results are reported in nmol of IMP formed per hour and per mg of hemoglobin. Hemoglobin concentration was determined in hemolysates using the Drabkin’s reagent.

### Determination of erythrocyte PRPP concentrations

The erythrocytes were separated by centrifugation at 1500×*g* for 10 min at 4 °C. The received pellet was washed three times with PBS and, after protein precipitation, PRPP concentrations were determined using a Precice^®^ kit (Novocib, Lyon, France). In the presence of Hyp, HGPRT converts PRPP to IMP which is further oxidized to XMP (xanthosine 5'-monophosphate) by downstream IMP dehydrogenase enzyme leading to simultaneous NADH_2_ formation. The amount of NADH_2_ formed is measured spectrophotometrically at 340 nm and is equivalent to the amount of PRPP in the assay.

### Density separation of red blood cells

The RBCs were separated according to their density using discontinuous iodixanol (OptiPrep^®^, Nycomed) density gradients [28]. Five iodixanol layers (2 mL each), with densities of 1.075, 1.085, 1.095, 1.105 and 1.115 g/mL, were carefully layered on top of each other in a test tube, with the densest one at the bottom. One ml of whole blood was carefully layered on top of the least dense layer and the tube was centrifuged at 2500×*g* for 25 min at 22 °C. Each layer was carefully obtained separately. The density distribution of RBCs was estimated by determining the hemoglobin concentration in each layer. Because the erythrocyte density increases as a function of age [29], old cells accumulated at the bottom (dense) layers of iodixanol.

### Statistical analysis

Statistical analyses were performed using STATISTICA (data analysis software system), version 10 software (StatSoft, Inc., 2011). Distributions were examined using a Shapiro–Wilk test which showed that some variables departed from a normal distribution (they were log-normal; values were reported as median and *Q*_25_–*Q*_75_).

Significance level of differences observed between analyzed time points (before exercise vs. after exercise vs. 30 min after exercise) for each participant was calculated using Friedman’s ANOVA with Dunn’s test for post hoc analysis. The significance level of differences observed between groups (untrained vs. trained) was calculated using the Mann–Whitney U test.

The accepted level of significance was defined as *P* < 0.05.

In order to demonstrate whether the observed correlations were statistically significant, Spearman’s rank correlation coefficient was applied.

## Results

The study enrolled 15 sedentary young men aged 23.1 ± 3.41 years and 15 rowers aged 24.3 ± 2.56 years with a BMI between 21.5 ± 2.51 kg/m^2^ (trained) and 22.5 ± 2.32 kg/m^2^ (sedentary). Immediately before the exercise test, heart rate and blood pressure measurements were performed. The values obtained were within normal ranges and did not differ significantly between the two treatment groups. In the group of sedentary subjects, the time of exercise test was 19.3 ± 4.55 min, whereas in that of trained subjects, 23.0 ± 5.97 min. In trained subjects, oxygen consumption at maximum load (52.8 ± 4.54 mL/kg/min) was significantly higher (*P* < 0.001) compared with the sedentary ones (43.2 ± 5.20 mL/kg/min; Table 1).

The effect of exercise on pH, venous blood gases, inorganic phosphorous (Pi) and lactate concentration (LA) is presented in Table 2.

**Table 2:** The effect of exercise on venous blood gases, inorganic phosphorus and lactate concentration

	Sedentary group			Trained group		
	Rest	Post-exercise	30-min recovery	Rest	Post-exercise	30-min recovery
pH	7.4 ± 0.06	7.2 ± 0.05**	7.4 ± 0.04	7.4 ± 0.06	7.2 ± 0.08*	7.4 ± 0.04
pO_2_ (mmHg)	31.8 ± 8.03	42.3 ± 7.15	32.5 ± 7.03	32.0 ± 7.89	44.3 ± 7.25	31.8 ± 7.13
pCO_2_ (mmHg)	42.3 ± 1.20	35.2 ± 1.82*	41.3 ± 0.9	42.8 ± 1.19	36.8 ± 1.62*	41.4 ± 0.89
${\text{HCO}}_{3}^{ - }$ (mmol/L)	23.3 ± 0.90	18.7 ± 1.81**	22.1 ± 1.21	23.2 ± 0.88	19.6 ± 1.61**	22.8 ± 1.11
Pi in plasma (mmol/L)	1.21 ± 0.41	1.42 ± 0.78^#^	1.41 ± 0.64^#^	1.14 ± 0.39	1.45 ± 0.63^#^	1.37 ± 0.58^#^
Pi in RBC (mmol/L)	1.11 ± 0.21	1.39 ± 0.20*	1.17 ± 0.16	1.16 ± 0.14	1.32 ± 0.22*	1.17 ± 0.14
LA (mmol/L)	1.4 ± 0.45	10.1 ± 2.76***	4.6^##,§^ ± 1.66	1.7 ± 0.37	9.8 ± 2.65***	3.6 ± 1.25^##^,^§^

Values are given as mean ± SD

*pO*_*2*_ oxygen tension, *pCO*_*2*_ carbon dioxide tension, $HCO_{3}^{ - }$ bicarbonate, *Pi* inorganic phosphorous, *LA* lactate

* *P* < 0.05; ** *P* < 0.01; *** *P* < 0.001; different from rest and 30-min recovery

^#^ *P* < 0.05; ^##^ *P* < 0.01; different from rest

^§^ *P* < 0.001; different from post-exercise

Immediately after the physical exercise, a significant decrease was observed in pH (*P* < 0.01) and pCO_2_ (*P* < 0.05), as well as in the concentration of ${\text{HCO}}_{3}^{ - }$ (*P* < 0.01), in both treatment groups without significant differences between them. At 30 min of recovery, pH and pCO_2_, as well as the concentration of ${\text{HCO}}_{3}^{ - }$, did not differ significantly from the values being observed before the exercise (Table 2).

Immediately after exercise, a significant (*P* < 0.05) increase, being maintained at 30 min of recovery, in Pi concentration in plasma was shown, too. The Pi concentration in RBCs was significantly higher (*P* < 0.05) after the physical exercise only (Table 2).

An approximately five-fold increase in blood LA concentration (*P* < 0.001) in both treatment groups was also demonstrated immediately after exercise without significant differences between sedentary and trained subjects. At 30 min of recovery, blood lactate concentration significantly decreased (*P* < 0.001) compared with the post-exercise value but was still significantly higher (*P* < 0.01) compared with the rest values (Table 2).

The study showed a significantly higher (*P* < 0.01) concentration of 2,3-BPG in RBCs in trained subjects (2.3 ± 0.18 µmol/mL) than in the sedentary ones (1.85 ± 0.21 µmol/mL; Table 1).

**Fig. 1:**
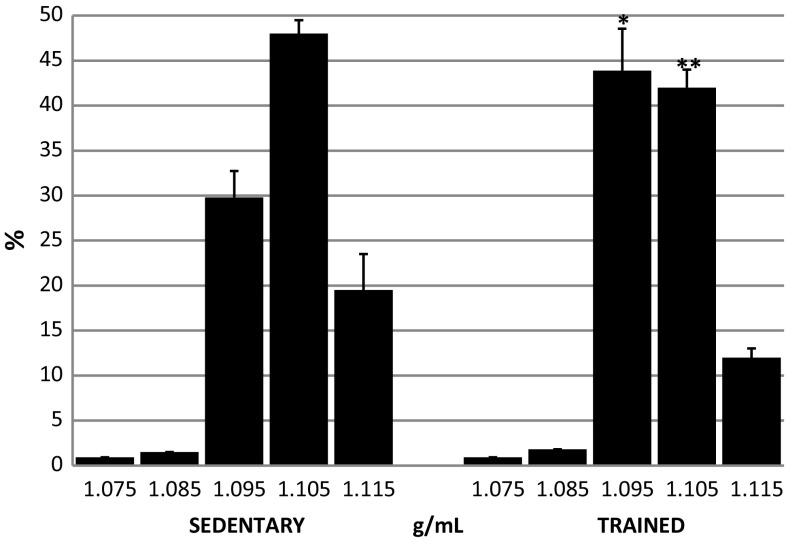
Percentage of RBCs distributed in density fractions between 1.075 and 1.115 g/mL in sedentary and trained subjects. *P < 0.05; **P < 0.01; different from sedentary

The density distribution of RBCs in the sedentary and trained groups is presented in Fig. 1. In both treatment groups, most RBCs accumulated in the layers with densities between 1.096 and 1.105 g/mL. In the layer of density 1.095 g/mL (where younger erythrocytes accumulate), sedentary and trained erythrocytes were 29.8 ± 2.94 and 43.9 ± 4.66%, respectively (*P* < 0.01). In the layer of density 1.105 g/mL, sedentary and trained erythrocytes were 48.0 ± 1.59 and 42.1 ± 2.06%, respectively (*P* < 0.001). In the layers of densities 1.075, 1.085 and 1.095 g/mL, where younger erythrocytes were accumulated, the percentage of erythrocytes from sedentary subjects was 32.2%, whereas in the trained ones, it was 46.6%.

**Fig. 2:**
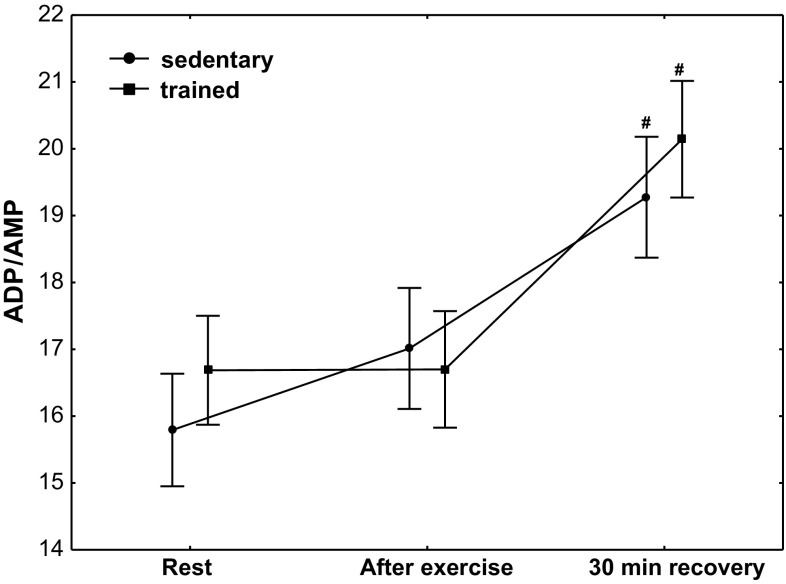
Erythrocyte ADP/AMP ratio at rest, after exercise and 30 min of recovery in sedentary and trained subjects. ^#^ *P* < 0.01; different from rest and after exercise

**Fig. 3:**
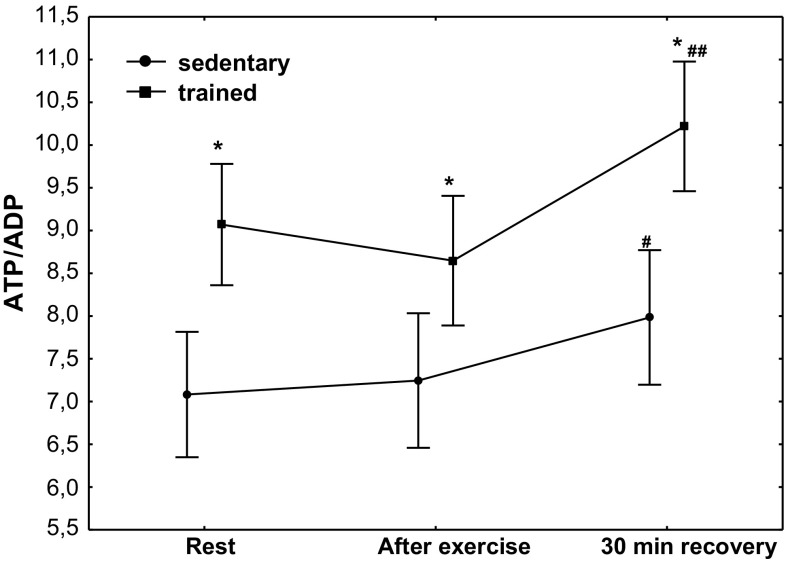
Erythrocyte ATP/ADP ratio at rest, after exercise and 30 min of recovery in sedentary and trained subjects. ^#^ *P* < 0.05; ^##^ *P* < 0.01; different from rest and after exercise. *P < 0.001; different from sedentary

The effect of physical exercise on purine nucleotide concentration in the group of sedentary and trained subjects is presented in Table 3. In the group of trained subjects, a significantly higher (*P* < 0.01) concentration of ATP was demonstrated at rest, after exercise and at 30 min of recovery compared with the sedentary group, whereas in both treatment groups, no post-exercise changes in ATP concentration were observed; the concentration of ADP and AMP significantly decreased (*P* < 0.01) at the 30th minute of recovery without significant differences between sedentary and trained subjects (Table 3). A significant decrease in ADP and AMP was accompanied by a significant increase (*P* < 0.01) in the value of AEC without changes in TAN (Table 3) and a significant increase in the ADP/AMP ratio (Fig. 2; *P* < 0.01). Although erythrocyte ATP/ADP ratios significantly increased in both groups (at the 30th minute of recovery), the ATP/ADP ratio was significantly higher (*P* < 0.001) in trained subjects compared with the sedentary ones (Fig. 3). In both treatment groups, a significant (Table 3; *P* < 0.01) post-exercise increase in IMP concentration in RBCs was observed (Table 3).

**Table 3:** The effect of exercise on purine nucleotide concentrations (ATP, ADP, AMP, IMP), TAN and AEC in red blood cells

Adenine nucleotides	Sedentary group			Trained group		
	Rest	Post-exercise	30-min recovery	Rest	Post-exercise	30-min recovery
ATP (µmol/L RBC)	1729 (1622–1741)	1746 (1794–1969)	1781 (1791–1989)	1875 (1862–1921)*	1883 (1794–1969)*	1879 (1791–1989)*
ADP (µmol/L RBC)	240 (238–250)	245 (237–250)	221 (217–231)^#^	220 (205–242)	218 (207–274)	199 (169–216)^**#**^
AMP (µmol/L RBC)	22.7 (18.45–24.82)	19.6 (16.98–22.54)	12.1 (12.37–17.68)^**#**^	20.6 (16.44–25.65)	18.3 (14.58–21.50)	9.8 (7.84–9.50)^**#**^
IMP (µmol/L RBC)	7.2 (7.40–9.82)	10.9 (8.28–11.04)^**§**^	7.9 (7.17–8.58)	7.9 (7.45–10.82)	11.6 (9.98–12.54)^**§**^	8.2 (7.37–9.68)
TAN (µmol/L RBC)	1991 (1892–2150)	2010 (1912–2025)	2014 (1814–2038)	2115 (2064–2126)	2119 (2021–2157)	2088 (2002–2150)
AEC	0.93 (0.93–0.94)	0.93 (0.93–0.94)	0.95 (0.94–0.95)^#^	0.94 (0.94–0.95)*	0.94 (0.94–0.95)*	0.95 (0.94–0.96)^#^

Values are given as medians (*Q*_25_–*Q*_75_)

*ATP* adenosine 5′-triphosphate, ADP adenosine 5′-diphosphate, *AMP* adenosine 5′-monophosphate, *IMP* inosine 5'-triphosphate, *TAN* total adenine nucleotide pool, AEC adenylate energy charge

* *P* < 0.01 different from sedentary subjects

^#^ *P* < 0.01 different from rest and after exercise

^§^ *P* < 0.01 different from rest and 30 min recovery

**Fig. 4:**
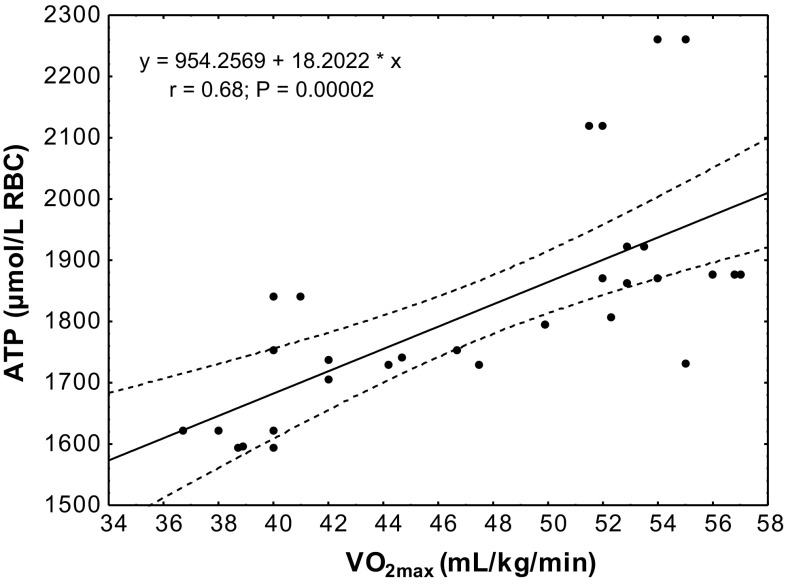
Correlation between erythrocyte ATP concentration and VO_2max_ being measured at rest

**Fig. 5:**
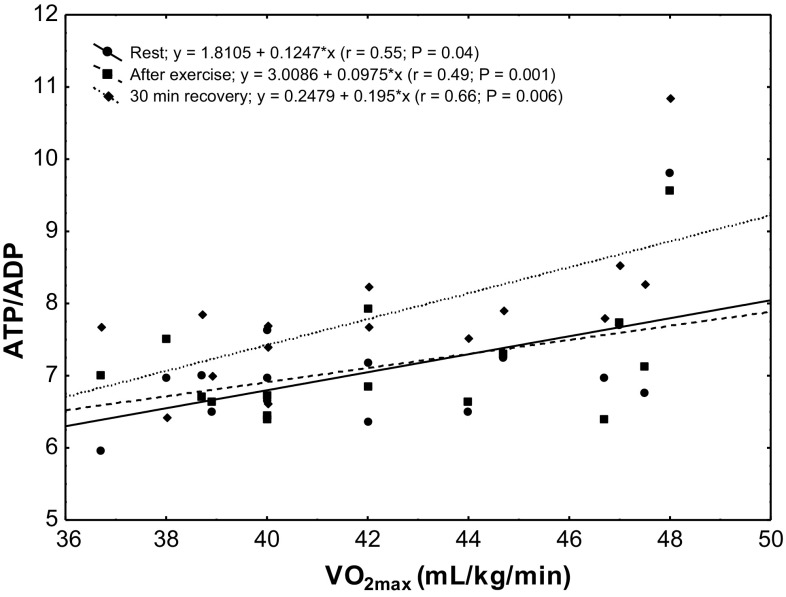
Correlation between erythrocyte ATP/ADP ratio and VO_2max_ in the sedentary group

**Fig. 6:**
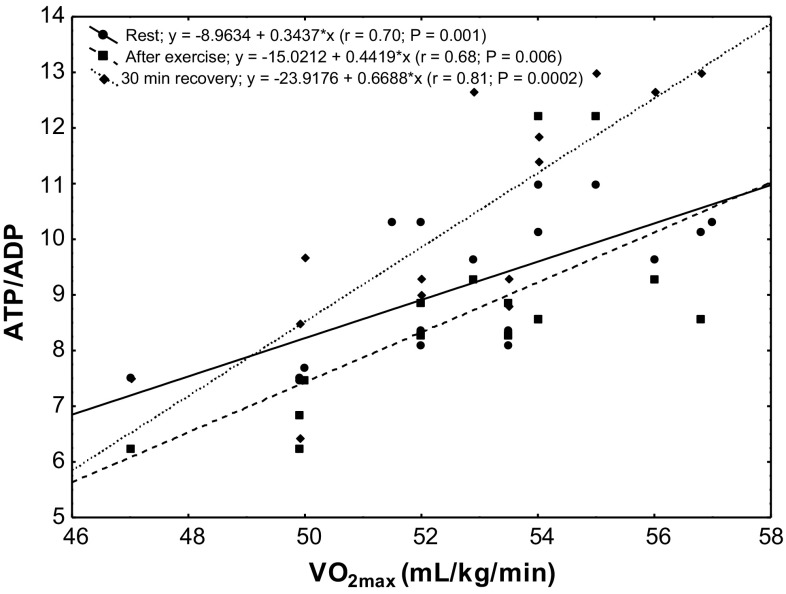
Correlation between erythrocyte ATP/ADP ratio and VO_2max_ in the trained group

The study showed a significant positive correlation (*r* = 0.68; *P* < 0.00002) between erythrocyte ATP concentration at rest and VO_2max_ level in the subjects (Fig. 4). In the group of untrained subjects, ATP/ADP ratio was correlated with VO_2max_ level at rest (*r* = 0.55; *P* < 0.04), after physical exercise (*r* = 0.49; *P* < 0.001) and in the 30th minute of recovery (*r* = 0.66; *P* < 0.006; Fig. 5). A similar correlation was observed in the group of trained subjects (rest *r* = 0.70, *P* < 0.001; after exercise *r* = 0.68, *P* < 0.006; 30 min of recovery *r* = 0.81, *P* < 0.0002; Fig. 6).

When evaluating the effect of high-intensity physical exercise in the activity of adenine nucleotide re-synthesis enzymes (Table 4), no post-exercise changes in APRT, HGPRT and PRPP-S activities and PRPP concentration were shown. However, significantly higher (*P* < 0.001) activities of APRT, HGPRT and PRPP-S and a significantly higher (*P* < 0.01) concentration of PRPP in RBCs were shown in the group of trained subjects compared with that of sedentary ones (Table 4).

**Table 4:** The effect of exercise on APRT, HPRT and PRPP-S activities and PRPP concentration in RBCs

	Sedentary group			Trained group		
	Rest	Post-exercise	30-min recovery	Rest	Post-exercise	30-min recovery
APRT (nmol/mgHb/min)	0.39 (0.35–0.43)	0.39 (0.38–0.42)	0.37 (0.33–0.42)	0.48 (0.48–0.57)**	0.44 (0.42–0.49)**	0.45 (0.42–0.52)**
HGPRT (nmol/mgHb/min)	1.68 (1.55–1.88)	1.77 (1.61–1.88)	1.68 (1.53–1.82)	1.91 (1.86–2.03)**	1.84 (1.85–1.93)**	1.87 (1.84–1.92)**
PRPP–S (nmol/mgHb/min)	1.23 (1.19–1.25)	1.23 (1.20–1.24)	1.19 (1.17–1.22)	1.32 (1.29–1.35)**	1.30 (1.22–1.35)**	1.31 (1.27–1.35)**
PRPP (µmol/L)	5.6 (4.90–5.90)	5.2 (5.24–6.70)	4.9 (4.86–5.52)	7.1 (6.52–7.85)*	7.0 (5.82–7.33)*	7.1 (6.91–7.4)*

Values are given as median (*Q*_25_–*Q*_75_)

*APRT* adenine phosphoribosyltransferase (EC 2.4.2.7), *HGPRT* hypoxanthine-guanine phosphoribosyltransferase (HGPRT; EC 2.4.2.8), *PRPP-S* 5-phosphoribosyl-1-pyrophosphate synthetase (EC 2.7.6.1), *PRPP* 5-phosphoribosyl-1-pyrophosphate

* *P* < 0.01; ** *P* < 0.001; different from sedentary subjects

**Fig. 7:**
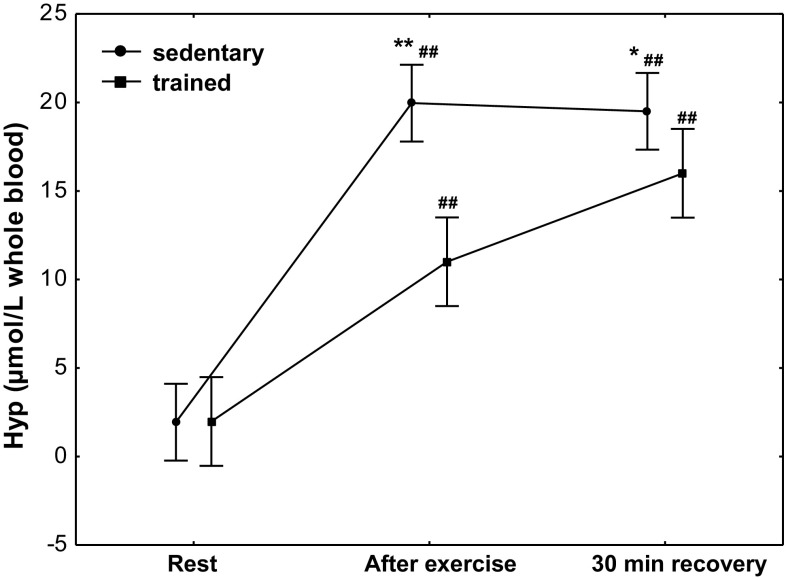
Blood hypoxanthine (Hyp) concentration at rest, after exercise and 30 min of recovery in sedentary and trained subjects. *P < 0.002; **P < 0.0001; different from trained. ^##^ *P* < 0.001; different from at rest

The concentration of Hyp (Fig. 7) at rest was 1.7 µmol/L whole blood (*Q*_25_–*Q*_75_ 0.11–4.04) in the group of trained subjects and 1.8 µmol/L whole blood (*Q*_25_–*Q*_75_ 0.19–3.91) in that of sedentary ones. After exercise, we observed a significant increase (*P* < 0.001) in Hyp concentration in both treatment groups to 11.1 µmol/L whole blood (*Q*_25_–*Q*_75_ 8.12–13.50) and 20.1 µmol/L whole blood (*Q*_25_–*Q*_75_ 17.46–22.52) in the groups of trained and sedentary subjects, respectively. A significantly higher Hyp concentration (*P* < 0.0001) was maintained at 30 min of recovery both in trained subjects (median 16.1 µmol/L whole blood; *Q*_25_–*Q*_75_ 13.50–18.35) and the sedentary ones (median 19.1 µmol/L whole blood; *Q*_25_–*Q*_75_ 16.80–21.67). A significantly higher increase in Hyp concentration after exercise (*P* < 0.0001) and at 30 min of recovery (*P* < 0.002) in the group of sedentary subjects was shown in the study (Fig. 7).

**Fig. 8:**
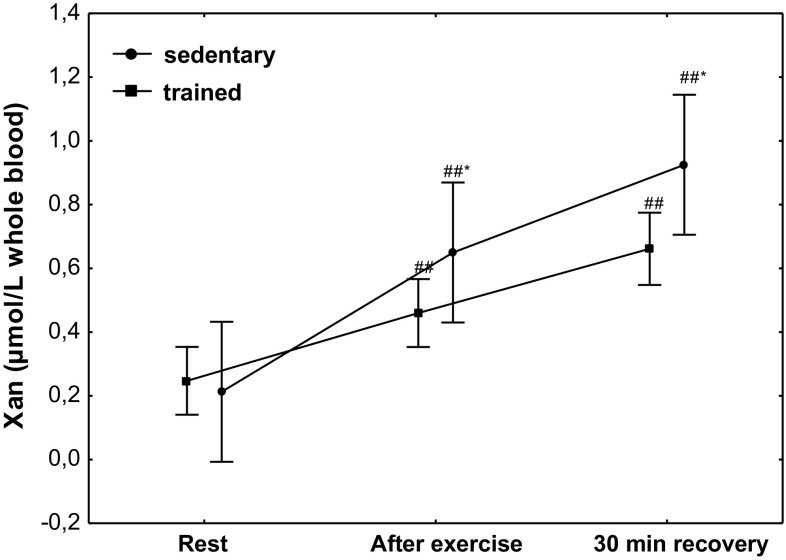
Blood xanthine (Xan) concentration at rest, after exercise and 30 min of recovery in sedentary and trained subjects. *P < 0.001; different from trained. ^##^ *P* < 0.001; different from at rest

The effect of physical exercise on Xan concentration is presented in Fig. 8. Compared to the at rest values, a significant increase (*P* < 0.001) in Xan concentration was observed in sedentary subjects, from 0.21 µmol/L whole blood (*Q*_25_–*Q*_75_ 0.03–0.45) to 0.65 µmol/L whole blood (*Q*_25_–*Q*_75_ 0.40–0.91), and in trained subjects (*P* < 0.001), from 0.26 µmol/L whole blood (*Q*_25_–*Q*_75_ 0.14–0.37) to 0.42 µmol/L whole blood (*Q*_25_–*Q*_75_ 0.33–0.55). Compared to the at rest values, a significantly higher (*P* < 0.001) concentration of Xan was maintained at 30 min of recovery in both treatment groups. A significantly higher increase in Xan concentration after exercise (*P* < 0.001) and at 30 min of recovery (*P* < 0.001) was observed in the group of sedentary subjects (Fig. 8).

**Fig. 9:**
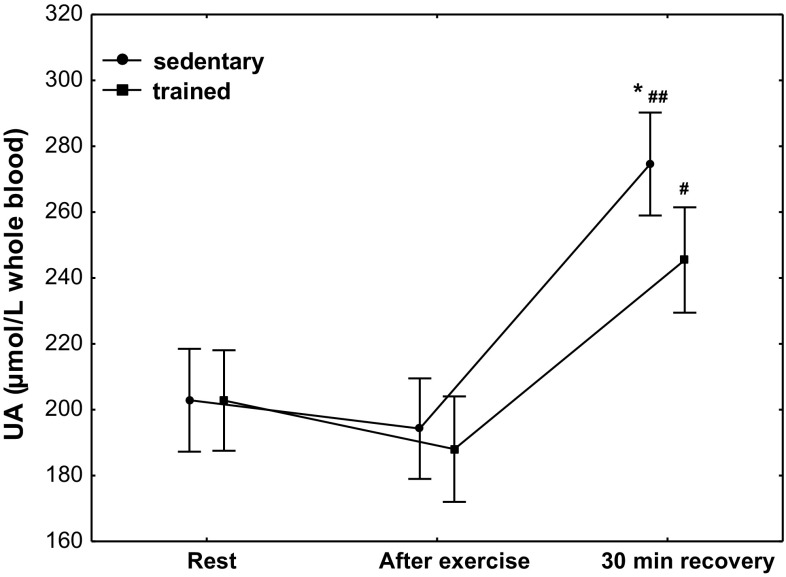
Blood uric acid (UA) concentration at rest, after exercise and 30 min of recovery in sedentary and trained subjects. *P < 0.05; different from trained. ^#^ *P* < 0.001; ^##^ *P* < 0.0001; different from rest and after exercise

The concentration of UA (Fig. 9) in the group of trained subjects at rest was 203 µmol/L whole blood (*Q*_25_–*Q*_75_ 189–218), while in that of sedentary ones, it was 202 µmol/L whole blood (*Q*_25_–*Q*_75_ 188–219). No significant changes in the concentration of UA were observed in both treatment groups. A significant increase in its concentration was observed at 30 min of recovery both in the group of trained subjects (*P* < 0.001; median 248 µmol/L whole blood) and that of sedentary ones (*P* < 0.0001; median 275 µmol/L whole blood), with a significantly higher increase in UA concentration (*P* < 0.05) at 30 min of recovery being observed in the group of sedentary subjects (Fig. 9).

**Fig. 10:**
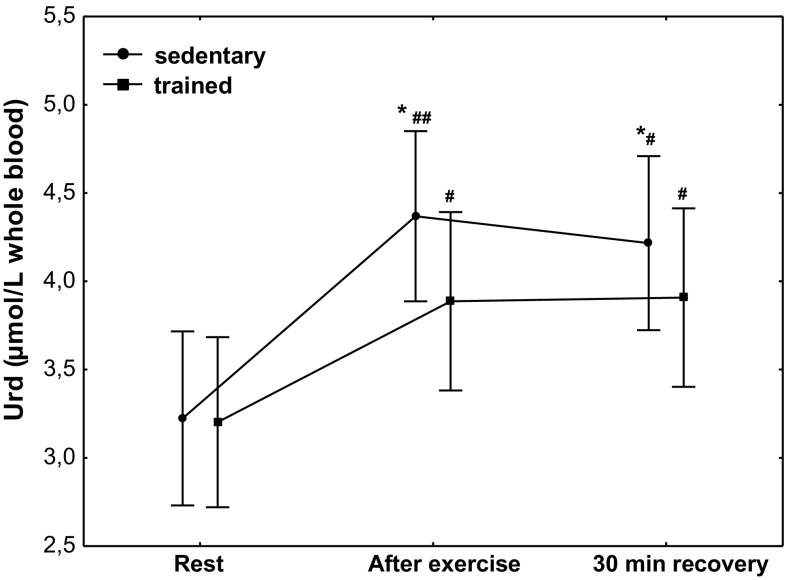
Blood uridine (Urd) concentration at rest, after exercise and 30 min of recovery in sedentary and trained subjects. *P < 0.05; different from trained. ^#^ *P* < 0.05; ^##^ *P* < 0.01; different from rest

The effect of physical exercise on the concentration of Urd is presented in Fig. 10. Compared to the at rest values, a significant increase (*P* < 0.01) in Urd concentration was observed in sedentary subjects, from 3.2 µmol/L whole blood (*Q*_25_–*Q*_75_ 2.65–3.70) to 4.4 µmol/L whole blood (*Q*_25_–*Q*_75_ 3.85–4.88), and in trained subjects (*P* < 0.05), from 3.1 µmol/L whole blood (*Q*_25_–*Q*_75_ 2.64–3.68) to 3.7 µmol/L whole blood (*Q*_25_–*Q*_75_ 3.38-4.38). Sedentary subjects had significantly higher post-exercise Urd concentration compared with the trained ones (*P* < 0.05). Compared to the at rest value, a significantly higher (*P* < 0.05) concentration of Urd was maintained at 30 min of recovery in both treatment groups, without significant differences between them (Fig. 10).

## Discussion

The study showed a significantly higher concentration of ATP in the erythrocytes of trained subjects which may at least partially reflect the increased re-synthesis of this nucleotide on the PRPP- and APRT-dependent pathway. This is supported by significantly higher PRPP concentration and APRT and PRPP-S activities in the erythrocytes of trained subjects. A relationship between PRPP synthesis rate and purine nucleotide biosynthesis rate regulation has been demonstrated previously [11, 30, 31]. It has been observed that APRT activity increases together with an increase in PRPP concentration [32, 33]. In human erythrocytes, PRPP concentration fluctuates around 1–5 µmol/L [11], just below the Km values of APRT and HGPRT (5–36 µmol/L) [31]. It is, therefore, possible that elevated levels of erythrocyte PRPP with increased PRPP-S and APRT activities would result in a higher synthetic rate of the purine salvage pathway, and could also explain the described high levels of ATP in the erythrocytes of trained subjects.

Our study also indicates that the differences being demonstrated in the concentration of analysed adenylates, as well as in the activity of analysed enzymes, may be in part related to the age of RBCs. In the study, RBCs were separated by density gradient centrifugation to evaluate the age distribution of erythrocytes. In comparison with the results of other authors [29, 34, 35], our study shows an increase in the proportion of young erythrocytes in the RBC population of trained subjects. Higher APRT activity has been observed in the erythrocytes of normal newborn infants [32, 33] which, as shown, may result in increased adenine nucleotide concentration in the RBCs of newborn infants. Similar differences have been shown between young and old erythrocytes being fractionated from the blood of adults. It is, therefore, possible that higher erythrocyte ATP concentration in athletes may be partially explained by an increase in the proportion of young erythrocytes. Young RBCs have an increased metabolic activity [36], higher 2,3-BPG and a lower Hb-O_2_ affinity than senescent RBCs [37, 38]. Our study demonstrated a significantly higher concentration of ATP in the erythrocytes of trained subjects and also, for the first time, a positive correlation between ATP concentration and physical capacity, being evaluated by a direct measurement of maximal oxygen uptake (VO_2max_). This allows us to state that erythrocyte ATP concentration is an important factor characterising physical capacity and exercise tolerance in healthy men. The presented study only allows us to speculate about the mechanisms through which ATP regulates physical capacity and VO_2max_. It is generally accepted that VO_2max_ during whole-body exercise is not constrained by mitochondrial oxygen consumption capacity but by the magnitude of oxygen delivery to the working muscles [21, 39]. Therefore, any factor(s) that improves oxygen delivery to the working muscles during physical exercise can contribute to the increased VO_2max_ and physical capacity. Recent studies have shown that erythrocyte ATP concentration is a major modulator of Hb affinity for oxygen. A rise in ATP leads to increased Hb-A p50 values, resulting, in turn, in reduced erythrocyte affinity for oxygen. A linear correlation between increasing ATP concentration and increases in p50 values of Hb-A has been observed [23]. It is, therefore, possible that better tissue oxygenation occurs together with an increase in ATP concentration in RBCs and a decrease in Hb affinity for oxygen and the same increases physical capacity and exercise tolerance. Thus, a significantly higher concentration of ATP in the erythrocytes of trained subjects may be an expression of favourable changes ensuring the adaptation of erythrocyte metabolism to increasing oxygen requirements of the body during physical exercise and training.

No significant post-exercise changes in ATP concentrations were observed in our study, whereas AMP and ADP concentrations in the 30th minute of recovery were significantly lower in the two groups of subjects, but differences between these groups showed no statistical significance. At the same time, a significant increase in the ATP/ADP and ADP/AMP ratios was also observed. The presented study thus suggests a post-exercise increase in the metabolism rate of ADP to ATP (significant increase in the ATP/ADP ratio). Although the physiological and clinical importance of the changes being observed is not clear, it is interesting that the erythrocyte ATP/ADP ratio shows a positive relationship with plasma nitric oxide (NO) concentration in healthy subjects (young and middle age), athlete subjects and diabetic patients [40]. It is, therefore, possible, and it will require further studies, that a post-exercise increase in the ATP/ADP ratio in RBCs contributes to the control of NO production and the regulation of blood flow. It has been shown in the in vitro studies that ATP is released from human RBCs and myocardium in response to short hypoxia periods [41]. The concentration of ATP has been shown to increase in response to incremental exercise in the coronary circulation of dogs exercising on a treadmill [42] and in the femoral vein in humans during knee extensor exercise [43, 44]. ATP being released from erythrocytes into the circulation can activate purinergic receptors, specifically those of the P_2Y_ subfamily, present on the vascular endothelium, resulting in the synthesis and release of NO [45, 46]. NO released abluminally interacts with the vascular smooth muscle, resulting in its relaxation and increased blood flow to hypoxic/exercising tissue [45, 47]. An original discovery of this study also is that the erythrocyte ATP/ADP ratio, as well as its exercise-induced increase, shows a significant positive correlation with VO_2max_, in both the trained and the untrained group. The correlation being demonstrated allows us to state that the erythrocyte ATP/ADP ratio, just as ATP, is one of many factors characterising exercise tolerance.

The increased Pi concentration in blood serum and RBCs being observed in our study after physical exercise could play a supportive role in the activation of PRPP-S because this enzyme has an absolute requirement for Pi for its activation [10, 11]. However, our study did not show any post-exercise changes in PRPP-S activity and PRPP concentration, or any changes in APRT and HGPRT activities. Similarly, Yamamoto et al. [12] have observed no changes in PRPP-S activity after moderate-intensity exercise in the athletes playing rugby football, when Pi concentration in blood plasma significantly increased from 1.12 ± 0.12 to 1.46 ± 0.22 mmol/L and in RBCs from 1.06 ± 0.10 to 1.33 ± 0.21 mmol/L. On the basis of the data presented above, one may speculate that if Pi indeed plays an important role in erythrocyte PRPP-S activity as reported by Berman [10] and Berman and Human [11], then the magnitude of exercise-induced changes in the blood Pi being observed in our study was too small or the duration of erythrocyte exposure to high plasma Pi was too short to cause a significant increase in PRPP-S activity.

An increase in IMP concentration in RBCs after high-intensity exercise has been already observed in our previous study in the group of young untrained men [18]. The present study confirms our previous results and, in addition, allows us to state that maximal physical exercise also results in an increase in IMP concentration in RBCs in the trained subjects. Because one of the routes of Hyp utilisation is its uptake by RBCs [48], a significant increase in blood Hyp concentration, as well as a significant increase in erythrocyte IMP concentration with no changes in ATP, ADP and AMP concentrations at the same time, being observed directly after physical exercise suggests that Hyp was included in the IMP pool in the reaction catalysed by HGPRT in the presence of PRPP. However, our study did not show any post-exercise changes in HGPRT and PRPP-S activities, indicating that Hyp utilisation by IMP in RBCs after physical exercise takes place with no changes in the activity of analysed enzymes. The in vitro studies have shown that incubation of RBCs in solutions with 7.35 and 7.25 pH and containing Pi at a 2-mmol/L concentration results in an increase in IMP concentration, whereas incubation in a solution with high Hyp level (50 µmol/L) without any changes in Pi concentration and pH induce no changes in IMP concentration [12]. In our study, an increase in IMP concentration was observed under increased Hyp concentration in blood and Pi concentration in plasma and erythrocytes. At the same time, a decrease in blood pH was also observed. These results suggest that an increase in IMP concentration in erythrocytes after maximal physical exercise is stimulated by a post-exercise increase in Pi concentration in erythrocytes. In the 30th minute of recovery, despite the persisting high concentration of Hyp in blood, an increase in IMP concentration was recorded no longer. At the same time, no changes in erythrocyte Pi concentration were observed. Thus, a decrease in pH and an increase in Pi and Hyp concentrations in blood being observed suggest that Hyp was included in the IMP pool in the reaction catalysed by HGPRT but without any changes in the post-exercise activity of this enzyme. An increase in IMP concentration in RBCs with no changes in erythrocyte HGPRT and PRPP-S activities was also observed after moderate-intensity exercise (65% VO_2max_) [12].

A post-exercise increase in Hyp, Xan and UA concentrations in blood has been already observed by many authors [6, 20, 49, 50]. Numerous studies have also documented that repeated high-intensity training decreases purine nucleotide losses and reduces the post-exercise flux of purines from muscles to blood [4, 20, 51, 52]. The results of our study are thus consistent with the literature data and confirm a significantly lower post-exercise increase in Hyp, Xan and UA concentrations in blood being observed in athletes. Increased ATP degradation, which accompanies high-intensity exercises, and a decrease in its concentration contribute not only to an increase in the concentration of purine degradation products (Hyp, Xan, UA) but also to reduced phosphorylation of UDP to UTP, leading to increased UDP and UMP levels. These changes accelerate pyrimidine degradation (UTP → UDP → UMP → Urd), which may result in an increase in the concentration of Urd in blood [53, 54]. Our previous studies have shown that high-intensity physical exercise leads to a post-exercise, and persisting in the 30th minute of recovery, increase in Urd concentration in blood [54, 55]. These studies have also shown that a post-exercise increase in Hyp concentration (myogenic purine degradation indicator) correlates with a post-exercise increase in Urd concentration in blood (myogenic pyrimidine degradation indicator) suggesting that the blood Urd level is related to skeletal muscle purine metabolism [54]. Although the results of the present study confirm our previous observations on a post-exercise increase in Urd concentration in blood [54, 55], they allow us, however, to state for the first time that there is a significantly lower post-exercise increase in Urd concentration in the trained subjects. Such an adaptation may have important consequences for the athletes performing high-intensity workouts as it is associated with reduction in the net losses of pyrimidine nucleotides, the participation of which in RNA synthesis, bio-membranes (via the formation of pyrimidine-lipid conjugates) and glutathione (via the formation of pyrimidine-sugar conjugates) is well-known and documented [56, 57].

Conclusion. Training results in favourable changes in purine and pyrimidine metabolism. These changes are expressed in a significantly higher concentration of ATP in the erythrocytes of trained subject, which may be partially explained by higher metabolic activity in the purine re-synthesis pathway, being typical for young RBCs. The ATP concentration, just as the ATP/ADP ratio, as well as an exercise-induced increase in this ratio, correlates with the VO_2max_ level in these subjects, allowing them to be considered as the important factors characterising physical capacity and exercise tolerance. Maximal physical exercise in the group of trained subjects results not only in a lower post-exercise increase in the concentration of Hyp, Xan and UA but also in that of Urd. This indicates the possibility of performing high-intensity work with a lower loss of not only purine but also pyrimidine. A single physical exercise event results in an increase in IMP concentration in RBCs without any changes in the post-exercise activity of HGPRT, PRPP-S and PRPP.
